# Significance of Novel Ideas to Solve Challenges Facing Today's Ophthalmology

**Published:** 2012

**Authors:** Fatemeh Heidary, Reza Gharebaghi

**Affiliations:** 1 School of Medicine, Shahid Beheshti University of Medical Sciences, Tehran, Iran

**Keywords:** Ophthalmology, Blindness, WHO, Low vision

**Potential threats**

Our world is caught in the terrible cycle of poverty, illiteracy, violence, and devastating disease. Countries have been subject to the weighty challenge inherent in addressing the recklessly growing population, pervasive unemployment, rising longevity, the burden of diseases, insurgencies, and political insecurity (1). Although healthcare systems have improved dramatically during recent years and new modalities of treatments have been introduced, still there are many challenges facing healthcare systems and in particular in ophthalmology and eye care services which require immediate reform.

**A dynamic science**

Ophthalmology has been progressing very quickly in recent decades, and is currently present in a reasonable number of surgery centers around the world. An enormous change has taken place in visual sciences, and despite the fact that no study has been made on measurement of growth of science in different specialty fields; ophthalmology might well be the discipline with the highest number of advancements. 

Presently, the new suture-less and noninvasive microsurgical techniques have made a revolutionary change, and the mortality as well as morbidity of various diseases has been reduced significantly as a result. Progress has been made in specific areas. For example, surgical techniques have made enormous development in both anterior and posterior ocular surgeries, and patients are able to resume their ordinary lives only few minutes after the surgeries. Post-operation complications have significantly decreased and the new generation of antibiotics has been introduced. However, there is much that still needs to be accomplished.

**Major ophthalmic challenges**

On the other hand, blindness and visual impairment act as significant and ubiquitous health problems. Although new technologies are rapidly progressing, visual impairment is still a noteworthy problem to worldwide healthcare systems. For that reason, international organizations have regulated that in the various universal projects, ophthalmic services as well as teaching facilities in visual sciences have to increase significantly. 

Currently, ophthalmology deals with major causes of blindness including cataracts, uncorrected refractive errors, glaucoma, age-related macular degeneration, corneal opacities, diabetic retinopathy, pediatric ocular diseases, trachoma and many infectious diseases. Although innovative treatment modalities have been presented well, so far no definitive treatment has been introduced for most of the chronic diseases. 

According 2012 world health organization fact sheets, 285 million people around the world are visually impaired. Specifically, 39 million are blind and 246 million have low vision. Universally, uncorrected refractive errors are one of the major reasons for visual impairment and cataracts remain the conspicuous reason for blindness in developing countries. The reality of it is that 80% of all visual impairment could be prevented or cured (2). Unfortunately, around 90 percent of visually impaired individuals live in developing countries. Global dissemination of visual impairment reveals a disproportionately large prevalence in low income countries. In developing countries, trachoma and cataracts are the largest causes of avoidable visual impairment and there is both a deficiency and inequity in accessibility of eye care. These are the major challenges facing today’s ophthalmologists worldwide.

On the other hand, effectiveness of off-label medications, presence of chronic diseases, safety of biological drugs, high cost of retinal procedures and intraocular lenses, legal issues in corneal donation, and economic instability in developing countries has caused a deferral of surgeries represent major problems that have yet to be resolved. 

There are also the numerous challenges that ophthalmic education has faced. Lack of specialized ophthalmic nurses to educate patients, lack of universal clinical guidelines to manage the cases in the uniform format, inability to teach residents in common ocular surgeries are the other problems in most developing countries. 

Inequality in distribution of ophthalmologists is one of the major problems in most areas. Private ophthalmic hospitals are one of the major threats to public health. Many cases have been shifted to private hospitals where the level of education has been compromised at the same time. In fact, there are many other serious problems we plan to discuss in future issues of the journal.

**Characteristics of this journal**

Medical Hypothesis, Discovery & Innovation (MEHDI) Ophthalmology Journal aims to create an environment that will cultivate publishing of important innovative ideas in solving the aforementioned problems facing to ophthalmology: hypotheses, and practice-related material, robust dialogue among innovative professionals, faster cycles of innovation, and wider distribution of advances in surgical techniques and instruments. 

Our goal is speed in publishing information and making it readily available in articles in various databases. Usually, in most scientific journals, manuscripts from well-known authors are routinely published as the author is a known commodity. However, we take the view that a fresh-minded, young author could have an idea that could conceivably change the direction of future researches.

We believe strongly that any hypothesis or idea might provide opportunities to improve life. Similarly, ideas that result in better allocation of human resources and/or access of people to healthcare and medical services are also anticipated. Therefore, we plan to make those ideas highly visible.

Together with the world health organization, international agency for the prevention of blindness, international non-governmental organizations, and the international ophthalmic community, we can all advocate for support to prevent and treat visual impairment and bring together novel ideas to solve challenges facing today's ophthalmology. Close coordination, as well as sublimation of ego and the natural desire to control, would be required. Meticulous coordination, regular communication, and unwavering commitment will be essential for success. Ophthalmologists in developed regions would have a precise role and responsibility in the accomplishment of Vision for the Future (3). Therefore, we welcome your contribution in all future issues. 

And finally, more care, more research and most of all more education are required if we are to combat the increasing rates of blindness and other visual impairments globally. Success is about teamwork and strong partners. Therefore, we ask your cooperation for a better future.

## DISCLOSURE

The authors report no conflicts of interest in this work.
